# Temporally ordered associations between type 2 diabetes and brain disorders – a Danish register-based cohort study

**DOI:** 10.1186/s12888-022-04163-z

**Published:** 2022-08-26

**Authors:** Theresa Wimberley, Henriette T. Horsdal, Isabell Brikell, Thomas M. Laursen, Aske Astrup, Giuseppe Fanelli, Janita Bralten, Geert Poelmans, Veerle Van Gils, Willemijn J. Jansen, Stephanie J. B. Vos, Valérie Bertaina-Anglade, Lucia Camacho-Barcia, Bernat Mora-Maltas, Fernando Fernandez-Aranda, Mònica B. Bonet, Jordi Salas-Salvadó, Barbara Franke, Søren Dalsgaard

**Affiliations:** 1 NCRR - National Centre for Register-based Research, Aarhus BSS, Aarhus University, Fuglesangs Allé 26, DK-8210 Aarhus V, Denmark; 2 CIRRAU - Centre for Integrated Register-based Research, Aarhus University, Aarhus, Denmark; 3 Department of Medical Epidemiology and Biostatistics, Karolinska Institutet, Stockholm, Sweden; 4 Department of Human Genetics, Radboud University Medical Center, Donders Institute for Brain, Cognition and Behaviour, Nijmegen, The Netherlands; 5 Department of Biomedical and Neuromotor Sciences, University of Bologna, Bologna, Italy; 6 Department of Psychiatry and Neuropsychology, School for Mental Health and NeuroScience, Alzheimer Centrum Limburg, Maastricht University, Maastricht, Netherlands; 7 Biotrial Neuroscience, Rennes, France; 8 Department of Psychiatry, University Hospital of Bellvitge, Barcelona, Spain; 9 Psychiatry and Mental Health Group, Neuroscience Program, Institut d’Investigació Biomèdica de Bellvitge-IDIBELL, Barcelona, Spain; 10 Consorcio CIBER, M.P. Fisiopatología de la Obesidad y Nutrición (CIBERObn), Instituto de Salud Carlos III (ISCIII), Madrid, Spain; 11 Department of Clinical Sciences, School of Medicine and Health Sciences, University of Barcelona, Barcelona, Spain; 12 Departament de Bioquímica i Biotecnologia, Universitat Rovira i Virgili, Reus, Spain; 13 Institut d’Investigació Sanitària Pere Virgili (IISPV). Hospital Universitari San Joan de Reus, Reus, Spain; 14 Departament de Bioquímica i Biotecnologia, Unitat de Nutrició Humana, Universitat Rovira i Virgili, Reus, Spain; 15 Department of Psychiatry, Radboud University Medical Center, Donders Institute for Brain, Cognition and Behaviour, Nijmegen, The Netherlands; 16 iPSYCH - The Lundbeck Foundation Initiative for Integrative Psychiatric Research, Copenhagen and Aarhus, Aarhus, Denmark; 17 Center for Child and Adolescent Psychiatry, Mental Health Services of the Capital Region, Copenhagen, Denmark; 18 Department of Clinical Medicine, University of Copenhagen, Copenhagen, Denmark

**Keywords:** Epidemiology, Insulin signaling, Neurological disorders, Psychiatric disorders, Temporally ordered analysis, Type 2 diabetes mellitus

## Abstract

**Background:**

Type 2 diabetes mellitus (T2DM) is linked with several neurodegenerative and psychiatric disorders, either as a comorbid condition or as a risk factor. We aimed to expand the evidence by examining associations with a broad range of brain disorders (psychiatric and neurological disorders, excluding late-onset neurodegenerative disorders), while also accounting for the temporal order of T2DM and these brain disorders.

**Methods:**

In a population-based cohort-study of 1,883,198 Danish citizens, born 1955–1984 and followed until end of 2016, we estimated associations between T2DM and 16 brain disorders first diagnosed between childhood and mid-adulthood. We calculated odds ratios (OR) and hazard ratios (HR) with 95% confidence intervals (CI) in temporally ordered analyses (brain disorder diagnosis after T2DM and vice versa), adjusted for sex, age, follow-up, birth year, and parental factors.

**Results:**

A total of 67,660 (3.6%) of the study population were identified as T2DM cases after age 30 and by a mean age of 45 years (SD of 8 years). T2DM was associated with most psychiatric disorders. Strongest associations were seen with other (i.e. non-anorectic) eating disorders (OR [95% CI]: 2.64 [2.36–2.94]) and schizophrenia spectrum disorder (2.73 [2.63–2.84]). Among neurological disorders especially inflammatory brain diseases (1.73 [1.57–1.91]) and epilepsy (1.67 [1.60–1.75]) were associated with T2DM. Most associations remained in both directions in the temporally ordered analyses. For most psychiatric disorders, associations were strongest in females.

**Conclusions:**

T2DM was associated with several psychiatric and neurological disorders, and most associations were consistently found for both temporal order of disorders. This suggests a shared etiology of T2DM and those brain disorders. This study can form the starting point for studies directed at further elucidating potential causal links between disorders and shared biological mechanisms.

**Supplementary Information:**

The online version contains supplementary material available at 10.1186/s12888-022-04163-z.

## Background

Type 2 diabetes mellitus (T2DM) is a chronic metabolic disease with a rising burden worldwide, particularly in Western Europe. In Denmark, the prevalence has increased from 1.2% in 1996 to 4.5% in 2016 [1], with a decrease in the incidence rate during the period 2011–2014, a trend also found in other studies [2–4]. T2DM typically onsets during late adulthood, but over the past decades the prevalence and incidence have particularly increased among adults aged 40–50 years compared with older age groups [1]. The etiology of T2DM includes lifestyle risk factors, such as obesity and low physical activity, but also an increased genetic liability to T2DM and psychiatric disorders [5, 6]. Across the lifespan, T2DM is associated with increased risks for adverse health outcomes, such as cardiovascular diseases, and premature death [7–9]. Studies have also found associations between T2DM and disorders of the brain, including vascular dementia and Alzheimer’s disease [10], substance use disorder, schizophrenia, bipolar disorder, depression, personality disorder, and attention-deficit/hyperactivity disorder (ADHD) [11–17], obsessive-compulsive disorder (OCD) [18], cognitive impairment [19], and also of filling a prescription for psychotropic drugs, including antidepressants, antipsychotics, and anxiolytics [20]. Recently, the term “insulinopathies of the brain” has been suggested to characterize these associations between T2DM and brain disorders [21, 22]. It should be noted that from here and onwards we use the term brain disorder when referring to a broad range of specific psychiatric and neurological disorders, although late-onset neurodegenerative disorders were not assessed in the present study.

To better understand the mechanisms underlying the co-occurrence of T2DM and brain disorders, the temporal order of disorder occurrence within an individual is important. Most of the previous studies, however, were cross-sectional or did not examine the temporal order of T2DM and the brain disorder bi-directionally. Estimating associations in both directions could provide additional clues about underlying mechanisms [23]. If an association is bi-directional, with comparable incidence rates of the brain disorder before and after T2DM, this may suggest that both disorders serve as independent risk factors for the onset of each other, or that the association is mainly driven by shared risk factors [24], such as unhealthy lifestyles [25] and common genetic risks [26, 27]. Previous studies have also suggested sex differences in the association between T2DM and brain disorders, though findings have been mixed [14, 28, 29], and more data on this is needed. In this study, we will add new knowledge on the sex-specific temporally ordered associations between T2DM and brain disorders.

## Methods

### Aims

To 1) estimate associations with T2DM for a broad range of both psychiatric and neurological disorders, 2) perform temporally ordered analyses to explore whether timing of the disorders influences the strength of the associations, and 3) estimate sex differences in these associations.

### Design

Cohort study based on governmental data collected prospectively for administrative purposes and stored in nationwide Danish registers, accessible to researchers through Statistics Denmark.

### Setting

#### Data sources

The Danish registers contain individual-level information on all Danish citizens, with each register capturing a different set of medical or socio-demographic information. The Danish Civil Registration System established in 1968 holds information on sex, date of birth and death, continuously updated information on place of living, and enables linkage with parental information [30]. Information on hospital contacts and diagnoses (main and supplementary) was available in the Danish National Patient Register and the Danish Psychiatric Central Research Register, which include inpatient contacts since 1977 and 1969, respectively, as well as outpatient and emergency room contacts since 1995 [31]. Diagnoses were classified according to the International Classification of Disease version 8 (ICD-8 [32]) until 1993, and version 10 (ICD-10 [33]) from 1994 onwards. Medication information was retrieved from the Danish National Prescription Registry, which includes information on all prescriptions redeemed at a Danish pharmacy since 1995 [34]. Information on education was available in the Education Registers at Statistics Denmark [35]. All the registers include an anonymized version of the unique personal identification number assigned to everyone at birth or immigration, which enables linkage across the registers on the individual level.

#### Study population

We included all individuals born in Denmark between 1955 and 1984, who were alive and residing in Denmark at age 30 years and could be linked to both parents. This cohort was chosen to enable identification of registered diagnoses of disorders occurring both early (childhood/adolescence) and later in life (adulthood) in the same individuals.

### Definition of T2DM

Date of first T2DM status was defined either with a clinical diagnosis of T2DM or the prescription of an oral antidiabetic drug (OAD, ATC codes A10B), whichever appeared first in the register after the age of 30 years. Additionally, some cases were identified based on having a hospital diagnosis of a T2DM-related complication as previously suggested [36]. For ICD-8 and ICD-10 codes, see Supplementary Table 1. To minimize misclassification of type 1 diabetes mellitus as T2DM cases, individuals only diagnosed or treated with OADs before age 30 years (but not later) were not included in our definition, inspired by previous register-based research [36]. In line with previous research [1], we ignored metformin prescriptions in females under age 40 years, as these could likely have been prescribed for polycystic ovarian syndrome. Women were thus defined as T2DM at first appearance of the other above-mentioned criteria after age 30 or at first redeemed metformin prescription after age 40.

### Definitions of brain disorders

Brain disorders were defined based on ICD disease categories with a sufficient number of cases both before and after T2DM in the same study cohort reaching a maximum age of 62 years, and hence did not include late-onset neurodegenerative disorders. Thus, we included the following psychiatric and neurological disorders: 1) psychiatric disorders: Obsessive-compulsive disorder (OCD), autism spectrum disorder (ASD), anorexia nervosa, other eating disorders, substance use disorder, schizophrenia spectrum disorders, major depressive disorder, bipolar disorder, anxiety disorders, personality disorders, and ADHD; 2) neurological disorders: Inflammatory brain diseases (including infections), amyotrophic lateral sclerosis (ALS), epilepsy, multiple sclerosis, and migraine. Information on ICD-8 and ICD-10 codes and data source for the diagnostic categories can be found in Supplementary Table 1.

### Covariates

The incidence of T2DM and the brain disorders we study differs between females and males, and hence analyses were adjusted for sex. To account for changes in the incidence of the disorders over time, analyses were adjusted for birth year. Psychiatric disorders and T2DM in parents were included as potential confounders, and measured at the time the individual reached age 30 years (baseline), in line with a previous study [16]. Parental psychiatric disorder were identified if at least one parent had a registered diagnosis in the Danish Psychiatric Central Research Register. Parental T2DM was based on diagnoses only (Supplementary Table 1), due to low coverage of prescription data in the parental generation.

### Statistical analysis

We used different approaches to study the association between T2DM and the brain disorders of interest. First, we estimated the overall association between the brain disorder of interest and T2DM by the odds ratio (OR) from logistic regression (i.e., the temporal order of the two was not considered in this analysis). We adjusted for the variation in length of follow-up between individuals by including the age at end of follow-up as a continuous covariate in the model.

Second, in temporally ordered analyses, we estimated hazard ratios (HRs) from Cox regression analyses for the association between the brain disorder and subsequent diagnosis of T2DM and vice versa, using age as the underlying time scale. Individuals were followed from age 30 years and until either the date of outcome, or censored at date of emigration from Denmark, death, or end of the study period (December 31, 2016). The exposure of interest (i.e., the brain disorder or T2DM) was included time-dependently after age 30 years. When we considered T2DM as the exposure and a brain disorder as the subsequent outcome, the study cohort excluded individuals with a previous diagnosis of the brain disorder of interest before age 30 years.

Third, due to sex differences in the incidence of brain disorders and T2DM, we performed analyses for males and females separately. All analyses were adjusted for the following confounders: Sex (except those stratified by sex), birth year, and parental diagnoses of T2DM and any psychiatric disorder, prior to the date the individual reached age 30 years.

In supplementary analyses, we included education as an additional covariate in the models, to examine the influence of (a proxy of) socio-economic status on the association. This was defined as the highest attained level of education of the individual by age 30 years, categorized as: Not completed primary school, low (primary school), intermediate (high school/vocational training), and high level (higher education). Moreover, we repeated the logistic regression analyses with two alternative register-based T2DM definitions: 1) Restricting to at least two OAD prescriptions as have been used in previous research [1], and 2) restricting to hospital diagnoses to evaluate the robustness of results across severity level of T2DM.

Proportional hazards were checked by log-minus-log survival plots. All estimates are accompanied by 95% confidence intervals (CIs). All analyses were conducted in Stata version 16 (StataCorp, College Station, Tex.).

## Results

### Descriptive statistics

The study cohort included a total of 1,883,198 individuals, of whom 48.3% were females and the mean age at the end of follow-up was 46.6 years (standard deviation (SD) 8.4). During follow-up 5.4% of the study population emigrated from Denmark and 2.8% died. We identified 67,660 (3.6%) individuals as T2DM cases with a mean age of 45.1 years (SD = 7.7) at first presentation according to our register-based definition. Number of cases for the different brain disorders and distributions of these by sex, age at diagnosis and end of follow-up, and parental history of T2DM and psychiatric disorders can be found in Table 1.

**Table 1:** Characteristics of the full cohort and of individuals with specific psychiatric and neurological disorders

	Brain disorder cases^a^ n (%)	Type 2 diabetes cases^b^ n (%)	Female sex n (%)	Age (years) at first diagnosis Mean (SD)	Age (years) at end of follow-up^c^ Mean (SD)	Parental history of psychiatric disorder n (%)	Parental history of type 2 diabetes n (%)
Total study population (*n* = 1,883,198)	–	67,660 (3.6)	909,341 (48.3)	–	46.6 (8.4)	260,463 (13.8)	102,382 (5.4)
**Psychiatric disorders**							
OCD	6856 (0.4)	327 (4.8)	4209 (61.4)	45.1 (7.7)	42.7 (7.8)	1621 (23.6)	504 (7.4)
ASD	3594 (0.2)	184 (5.1)	1145 (31.9)	32.9 (9.4)	42.0 (7.8)	834 (23.2)	244 (6.8)
Anorexia nervosa	4846 (0.3)	95 (2.0)	4592 (94.8)	30.5 (12.4)	41.8 (7.6)	1023 (21.1)	275 (5.7)
Other eating disorder	7646 (0.4)	354 (4.6)	7286 (95.3)	23.8 (8.6)	40.6 (6.6)	1694 (22.2)	579 (7.6)
Substance use disorder	138,410 (7.3)	9456 (6.8)	49,432 (35.7)	27.8 (8.6)	46.4 (8.5)	33,167 (24.0)	9564 (6.9)
Schizophrenia spectrum disorder	33,603 (1.8)	3082 (9.2)	14,037 (41.8)	33.0 (11.5)	46.4 (8.5)	9704 (28.9)	2389 (7.1)
Major depressive disorder	73,497 (3.9)	5048 (6.9)	44,532 (60.6)	30.5 (9.4)	46.1 (8.3)	17,849 (24.3)	4997 (6.8)
Bipolar disorder	12,397 (0.7)	936 (7.6)	7056 (56.9)	36.2 (9.5)	47.2 (8.4)	3535 (28.5)	691 (5.6)
Anxiety	47,922 (2.5)	2906 (6.1)	28,556 (59.6)	37.1 (9.8)	45.2 (8.2)	12,291 (25.6)	3497 (7.3)
Personality disorder	55,572 (3.0)	4075 (7.3)	32,715 (58.9)	35.2 (9.7)	45.6 (8.5)	15,798 (28.4)	3969 (7.1)
ADHD	10,523 (0.6)	443 (4.2)	4178 (39.7)	30.0 (8.8)	41.2 (6.8)	3138 (29.8)	969 (9.2)
**Neurological disorders**							
Inflammatory brain diseases	7777 (0.4)	424 (5.5)	3516 (45.2)	27.1 (16.6)	45.2 (8.4)	1296 (16.7)	490 (6.3)
Amyotrophic lateral sclerosis	544 (0.0)	28 (5.1)	209 (38.4)	44.5 (9.7)	50.0 (7.7)	64 (11.8)	25 (4.6)
Epilepsy	39,634 (2.1)	2238 (5.6)	18,445 (46.5)	26.9 (14.4)	46.0 (8.3)	7405 (18.7)	2733 (6.9)
Multiple sclerosis	9860 (0.5)	375 (3.8)	6672 (67.7)	36.9 (8.7)	48.0 (7.8)	1322 (13.4)	575 (5.8)
Migraine	35,799 (1.9)	1540 (4.3)	26,018 (72.7)	34.5 (11.0)	46.9 (8.0)	6070 (17.0)	2006 (5.6)

^a^Number of cases refer to those in the total study population during follow-up and not all cases are included in the separate analyses examining the temporal order of type 2 diabetes and brain disorder. Note that groups of brain disorders are not mutually exclusive, i.e. individuals may have several brain disorder diagnoses before end of follow-up

^b^Type 2 diabetes are only considered after age 30, i.e. individuals that were only diagnosed or treated with oral antidiabetics before age 30 were not included as type 2 diabetes cases

^c^Age at end of follow-up equals age at death. Emigration, or end of study, whichever comes first. Note that the varying age at end of follow-up is accounted for in the statistical analyses but not in the descriptive table

Abbreviations: ADHD: attention-deficit/hyperactivity disorder. ASD: Autism spectrum disorder. OCD: Obsessive-compulsive disorder

### Associations between brain disorders and T2DM

**Fig. 1:**
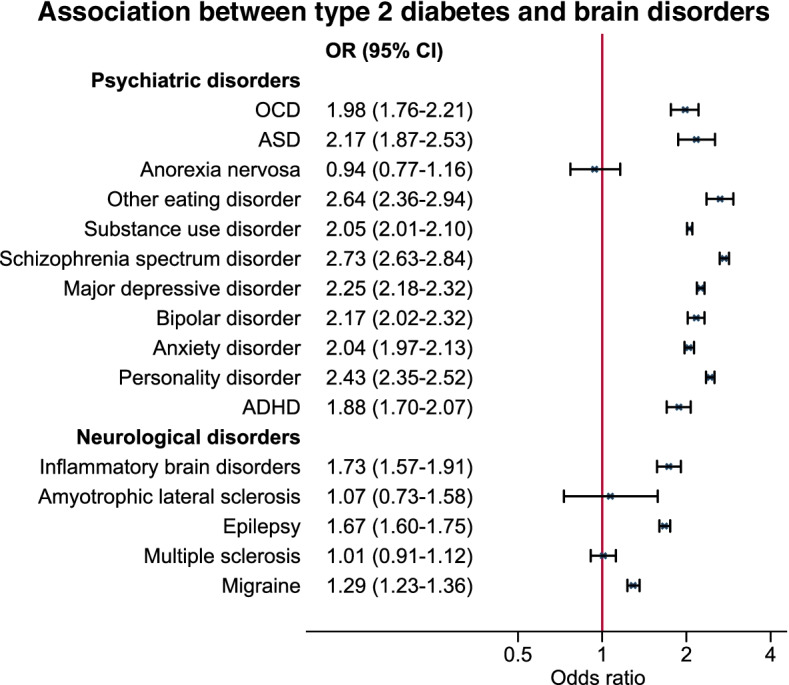
Adjusted odds ratios (ORs) and 95% confidence intervals (CIs) for the association between type 2 diabetes and included psychiatric and neurological disorders. Analyses were based on the entire study population of 1,883,198 individuals, and were adjusted for sex, birth year, age at end of follow-up, and parental history of psychiatric disorders and type 2 diabetes. Minimally adjusted estimates as well as number of exposed cases can be seen in Supplementary Table 2. Abbreviations: ADHD: attention-deficit/hyperactivity disorder, ASD: Autism spectrum disorder, OCD: Obsessive-compulsive disorder

Associations with T2DM were found for most of the brain disorders studied when not considering the temporal order of diagnoses. The associated brain disorders were OCD, ASD, eating disorders (except anorexia nervosa), substance use disorder, schizophrenia spectrum disorder, major depressive episode, bipolar disorder, anxiety, personality disorder, ADHD, inflammatory brain disease, and epilepsy. No clear evidence of an association was found for anorexia nervosa, ALS, or multiple sclerosis as reflected by adjusted ORs close to the null (0.94, 1.07, and 1.01, respectively) and the width of the accompanying 95% confidence intervals. Adjusted ORs are presented in Fig. 1, and the number of cases as well as crude and adjusted ORs are shown in Supplementary Table 2. Almost all psychiatric disorders were associated with T2DM. Strongest associations were observed for eating disorders other than anorexia nervosa (adjusted OR [95% CI]: 2.64 [2.36–2.94]) and for schizophrenia spectrum disorder (2.73 [2.63–2.84]). Among the neurological disorders, associations were found for inflammatory brain diseases (1.73 [1.57–1.91]), epilepsy (1.67 [1.60–1.75]), and migraine (1.29 [1.23–1.36]). Virtually no differences were observed in crude and adjusted estimates.

### Temporally ordered analyses

**Fig. 2:**
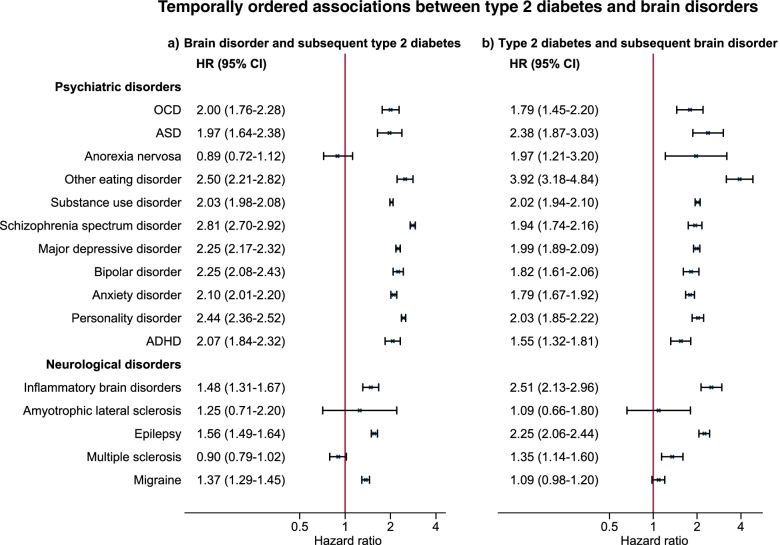
Adjusted hazard ratios (HRs) and 95% confidence intervals (CIs) based on temporally ordered analyses for the association between type 2 diabetes and included psychiatric and neurological disorders. Analyses were adjusted for sex, birth year, and parental history of psychiatric disorders and type 2 diabetes. **a** Individuals (*n* = 1,883,198) were followed for type 2 diabetes from age 30 until end of follow-up, and the brain disorder of interest were included as a time-dependent exposure variable after age 30, and time-fixed for disorders diagnosed before age 30. *N* = 1,883,198 **b** Individuals were followed for the brain disorder of interest from age 30 until end of follow-up, and type 2 diabetes status was included as a time-dependent exposure. For the last approach (type 2 diabetes and subsequent brain disorder), only incident cases were included and hence the total number of individuals included in each analysis varied for each outcome, e.g. for OCD as the outcome (*n* = 1,880,480) and for ASD as the outcome (*n* = 1,881,576). Minimally adjusted estimates as well as number of exposed cases can be seen in Supplementary Table 2. Abbreviations: ADHD: attention-deficit/hyperactivity disorder, ASD: Autism spectrum disorder, OCD: Obsessive-compulsive disorder

When examining the association between a brain disorder and subsequent T2DM, the estimates were very similar to those seen in the logistic regression, with increased rates of T2DM for all brain disorders, except for anorexia nervosa (adjusted HR [95% CI]: 0.89 [0.72–1.12]), ALS (1.25 [0.71–2.20]), and multiple sclerosis (0.90 [0.79–1.02]) (Fig. 2a).

When examining the opposite temporal order of the disorders, i.e. T2DM first and brain disorder later, the pattern was overall similar, though with some noticeable differences. T2DM was associated with all the examined psychiatric disorders, now also anorexia nervosa (adjusted HR [95% CI]: 1.97 [1.21–3.20]). Furthermore, among the neurological disorders, an association with later multiple sclerosis was also observed (1.35 [1.14–1.60]), while an association with later migraine was less clear as observed by a smaller estimated effect size with a 95% confidence interval overlapping 1 (1.09 [0.98–1.20]) (Fig. 2b).

Overall, the proportionality assumptions were fulfilled in most analyses. However, in the second approach (T2DM after brain disorder), proportionality was not fulfilled for anorexia nervosa, with rates indicating a positive association with the incidence of T2DM at earlier ages and a negative association in later years of follow-up.

### Sex differences in associations between T2DM and brain disorders

**Fig. 3:**
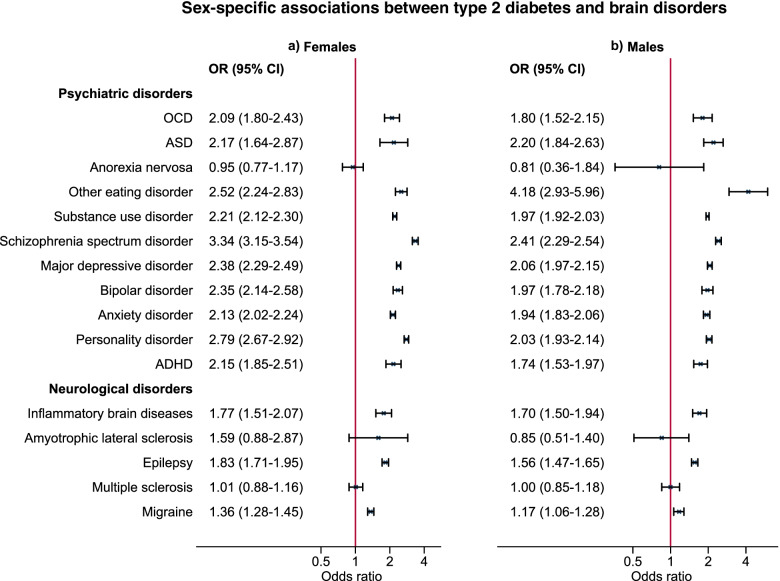
Adjusted odds ratios (ORs) and 95% confidence intervals (CIs) for the association between type 2 diabetes and included psychiatric and neurological disorders in females and males. Analyses were adjusted for birth year, age at end of follow-up, and parental history of psychiatric disorders and type 2 diabetes. Estimates were based on the total cohort of *N* = 1,883,198 individuals, and number of exposed cases as well as HRs for temporally ordered analyses can be seen in Supplementary Table 3. Abbreviations: ADHD: attention-deficit/hyperactivity disorder, ASD: Autism spectrum disorder, OCD: Obsessive-compulsive disorder

No significant sex differences were seen for OCD and ASD across the analytical approaches (Fig. 3 and Supplementary Table 3). For most other psychiatric disorders, associations were slightly stronger in females than in males. For anorexia nervosa, the adjusted OR was slightly below 1 in both females (0.95 [0.77–1.17]) and males (0.81 [0.36–1.84]) and with broad 95% confidence intervals, particularly for males. Numbers were too low to conduct sex-specific temporally ordered analyses. In contrast to most psychiatric disorders, the association between other eating disorders and T2DM was stronger in males (adjusted OR [95% CI]: 4.18 [2.93–5.96]) than in females (2.52 [2.24–2.83]) in logistic regression analyses but also in the temporally ordered analyses (Supplementary Table 3). Among the neurological disorders, estimates of the associations with epilepsy, and migraine were highest in females and confidence intervals did not overlap the estimates for males (Fig. 3 and Supplementary Table 3); for migraine diagnosed after T2DM, the association was less clear in females (adjusted HR [95% CI] = 1.18 [1.05,1.33]) and the adjusted HR was below 1 in males, but with 95% confidence limits overlapping 1 (0.87 [0.71–1.07]) .

### Influence of educational attainment and different definitions of T2DM

Associations estimated in the main models remained after further adjustment for highest attained level of education, though estimates were attenuated (Supplementary Table 4). Similarly, our estimates of the logistic regression analyses across different T2DM definitions were largely unchanged, though increased for several of the disorders when restricting to hospital diagnoses only (Supplementary Table 5).

## Discussion

In this most comprehensive cohort study of nearly 1.9 million adults, we investigated associations between T2DM and psychiatric and neurological disorders using different analytic approaches. In both the logistic regression (where the temporal order of disease occurrence was ignored) and in Cox regression models (temporally ordered analysis), we consistently found that T2DM was associated with the majority of the examined brain disorders. Out of the 16 disorders investigated, 15 showed significant association with T2DM in at least one of the analyses. This included a wide range of psychiatric disorders, as well as some neurological disorders, including inflammatory brain diseases, epilepsy and migraine. While most brain disorders were associated with T2DM irrespective of the temporal order of the two, T2DM was only associated unidirectionally with later diagnosed anorexia nervosa and multiple sclerosis, and the association with migraine was mainly evident, when considered prior to diabetes diagnosis, and we found no evidence of an association with ALS. For several of the psychiatric disorders (schizophrenia spectrum disorder, major depressive episode, bipolar disorder, anxiety, personality disorder, and ADHD), associations with subsequent T2DM were stronger than for incident diagnoses following T2DM.

The underlying mechanisms for associations between T2DM and brain disorders are largely unknown, though there is evidence for shared risk factors, both environmental and genetic [37]. Indeed, it has been hypothesized that the dysregulation of insulin signaling as observed in T2DM, metabolic syndrome, and obesity also extends to the brain in so-called “insulinopathies of the brain” [21]. Prior brain imaging studies offer some support for this concept, documenting gray matter changes in patients with T2DM, when compared with controls [38]. Also in recent genetic studies examining pathways contributing to compulsivity-related mental disorders and traits, insulin signaling has been identified as an important mechanism involved [39, 40].

### Temporal order of T2DM and brain disorders

Few previous studies have investigated the development of psychiatric disorders both before and after T2DM diagnosis. Such temporally ordered analyses of the bi-directional associations provide new additional knowledge about mechanisms involved in the comorbidity, as compared to cross-sectional or uni-directional analyses.

For the association of psychiatric disorders and subsequent T2DM, our results replicate previous findings of increased risk of T2DM in individuals with psychiatric disorders, including substance use disorder, schizophrenia, depression, bipolar disorder, personality disorder, and ADHD, all with similar effect sizes as ours, despite a younger cohort in our study and differences in T2DM definitions [13, 14, 17, 41]. Our study also adds to the literature by demonstrating significant positive associations of other specific psychiatric disorders, namely OCD, ASD, other (non-anorectic) eating disorders, and anxiety, with subsequent diagnoses of T2DM.

For the opposite temporal order of occurrence of diagnoses, previous studies have found that patients with T2DM had increased risk of subsequent schizophrenia and bipolar disorder [41], and of filling a prescription for psychotropic medications [20]. The findings for schizophrenia and bipolar disorder are replicated and extended by our study, showing that these effects are bidirectional. Our finding of a bidirectioncal relation between T2DM and major depressive disorder also corroborates previous findings [13]. Furthermore, our study adds to the literature with novel findings of bidirectional associations for other eating disorder than anorexia, substance use disorder, anxiety disorder, personality disorder, and inflammatory brain disorders. This may suggest that shared risk factors are at play. Indeed, recent data-driven genome-wide approaches indicate that T2DM and most of the psychiatric disorders share genetic risk factors [42]. Still, for several of the disorders, estimated effects were of different size in the two temporally ordered analyses, which could be explained by specific disease-related risk factors influencing the association in the one direction. Additionally, we found associations in both directions for T2DM with ASD and ADHD. However, these results should be interpreted with caution due to less register coverage and potential diagnostic delay for these childhood disorders.

The increased risk of eating disorders observed after T2DM may to some extent be explained by recommended changes in dietary patterns following diagnosis of T2DM. For anorexia nervosa we found that individuals previously diagnosed with T2DM also had an increased risk for later-diagnosed anorexia nervosa, but we did not find evidence for an association in the opposite direction. This may also suggest that those diagnosed with anorexia nervosa late in adulthood may differ from cases diagnosed during adolescence. Little is known about phenomenological differences in the two age groups of anorexia cases; from the existing literature, it is unclear whether a first diagnosis of anorexia nervosa in later adulthood truly represents late-onset of the disorder or simply diagnostic delay of a pre-existing disorder [43]. For the associations between T2DM and eating disorders other than anorexia nervosa, results from the temporally ordered analyses showed higher effect sizes for developing the eating disorder after T2DM (with a 4-fold increased risk) than for the opposite direction (where it was a 2-fold increased risk). One possible explanation of this could be, that late onset-eating disorders (other than anorexia nervosa) are associated with higher rates of premorbid obesity, when compared to those with the typical onset eating disorder subtype [44]. In addition, the duration of the eating disorder, the trajectory of body mass index (BMI) over time, and the eating disorder subtype seem to be crucial to shed some light on the nature of this relationship [45].

Many of our findings related to neurological disorders are in line with previous research. Studies have found epilepsy to be associated with later T2DM [46] and also vice-versa [47], and associations between T2DM and subsequent risk of inflammatory brain disorders [48]. In contrast, our finding of inflammatory brain disorders being associated with increased risk of developing T2DM is novel, as far as we know, though this was previously hypothesized in a review [49]. A previous study suggested a protective effect of T2DM on ALS in European individuals [50], while we did not find much evidence of an inverse association in any of our analyses, but number of ALS cases with T2DM was small as reflected by the broad confidence intervals.

Findings on associations between migraine and T2DM have suggested another pattern. We found that migraine was associated with increased risk of T2DM, but found less clear evidence for an association between T2DM and subsequent migraine. In line with our findings, a prospective survey of more than 70,000 women in France, found that prior migraine was associated with increased incidence of T2DM (HR = 1.16; 1.06–1.27), while current active migraine was associated with a lower risk of T2DM (HR = 0.80; 0.67–0.96) [51].

For multiple sclerosis, we also found an association only in one direction, i.e. that individuals with T2DM were at increased risk of later multiple sclerosis (but not vice-versa). One previous study also found increased risk of multiple sclerosis after T2DM, but did not examine the bi-directionality of the association [52]. Interestingly, there has been an increase in the incidence of especially late-onset multiple sclerosis over the past decades [53]. This trend coincides with increases in the incidence of T2DM [1]. Furthermore, previous research indicate a causal relationship between obesity and later multiple sclerosis [54]. Our finding that T2DM was only associated with later-onset multiple sclerosis may indicate a potentially causal link between T2DM or other insulin-related disorders and later development of multiple sclerosis, but this finding needs replication in different cohorts.

### Sex differences in associations between T2DM and brain disorders

Sex differences in the associations with T2DM were most pronounced for psychiatric disorders, with associations stronger in females than in males. These results are in line with previous research demonstrating stronger associations between OCD, anxiety, depression, bipolar disorder, and schizophrenia and T2DM in females, compared to males [18, 41, 55]. For migraine and epilepsy, we similarly observed stronger associations with T2DM in females, than in males. Interestingly, we did not find a statistically significant association between T2DM and subsequent risk of migraine in males, and the point estimate was even below 1 (HR = 0.87; 0.71–1.07). In a large nationwide register-based study from Norway, T2DM was associated with a reduced risk of subsequent migraine with a tendency to be more pronounced in males (HR = 0.82), than in females (HR = 0.91) [56]. In contrast, associations between T2DM and other (non-anorectic) eating disorders were stronger in males, compared with females, actually bidirectionally. A previous study from Finland also found that males with other eating disorders had higher risks of developing T2DM, than females [57]. This may partly be explained by a higher BMI in males, compared with females with other eating disorders [58]. Sex differences were less clear in the associations with subsequent depression, bipolar, anxiety, or personality disorder.

### Strengths and limitations

The major strength of the present study is the use of data from the nationwide registers, continuously updated for decades, and with virtually complete coverage. The clinical diagnoses are in general of good validity [31], though some diagnoses, such as OCD, have only been validated in children [59].

Our study also has several limitations. First, we only include data from hospital contacts, not diagnoses made by psychiatrists, neurologists, or general physicians in private practices. Hence, we may mainly capture the more severe cases, and our results may not be generalizable to milder cases. For T2DM, we reduced this potential bias by also including prescriptions of OADs, thereby capturing a large part of T2DM cases treated solely by general physicians. Our sensitivity analyses restricting to hospital-diagnosed T2DM confirms such a selection bias and potential ascertainment bias as associations were generally stronger compared to our main definition including prescription data. Still, misclassification by not correctly identifying all T2DM cases or by including some with type 1 diabetes cannot be ruled out. Second, as in other studies, the date of first diagnosis may not represent the onset of the disorder, e.g. first diagnosis of ASD or ADHD may be in adolescence or adulthood, although a childhood-onset is a diagnostic prerequisite for these disorders [60, 61]. Similarly, T2DM has a gradual onset, often asymptomatic initially and may also remain undiagnosed for several years [62]. Thus, such diagnostic delays may reduce the validity of some of the temporally ordered analyses and complicate interpretation of results from an etiological perspective. Particularly among females under age 40, the onset of T2DM may be biased by some delay, and incidence may be slightly underestimated, as we ignore potential cases of polycystic ovarian syndrome (those only prescribed metformin before age 40), which may be misclassified.

Third, the inclusion of only incident brain disorder cases in the temporally ordered analyses of T2DM and subsequent brain disorders may have led to an underestimation of these associations, because younger age of onset of psychiatric or neurological disorders is typically associated with more severe pathology. For some of the brain disorders, we identified few incident cases after T2DM diagnosis, and estimates should be interpreted with caution. Fourth, to study associations with both early and late diagnosis of brain disorders we used the same study population for all analyses, and hence only cases with a T2DM diagnosis before age 62 years were included. While this enabled us to estimate associations with brain disorders occurring early in life, a major part of later-diagnosed cases with T2DM were not included. Consequently, we could not estimate associations with late-onset neurodegenerative disorders, such as dementia and Alzheimer’s disease, associations which have been previously documented, also based on Danish registry data [10]. Similarly, information on mental disorders was not available from early childhood for individuals born in earliest calendar years. However, the incidence of such diagnoses during the 1970’s was extremely low, with an annual incidence of 17 per 100,000 children under age 15 years and 80% of those were readmitted after the age of 16 years [63]. For all study participants, we had complete coverage from age 14 years (or younger) and up to 32 years of coverage after age 30. Moreover, all analyses were adjusted for calendar year of birth and length of follow-up to minimize the potential influence of calendar time trends and differences in observation time between individuals. Finally, our observational data cannot inform about potential causality between T2DM and the development of brain disorders. Analyses were not adjusted for diverse variables such as BMI or other lifestyle factors including dietary intake or smoking, as such data was not available, and this may have influenced estimates towards a possible overestimation of the association. We did not develop unique adjustment models for each pairwise analysis, and we did not include information on the use of different types of medication (e.g. antipsychotics or antidiabetics). Rather, to keep analyses tractable and estimates comparable, we used the same adjustments across models and in both directions. Estimates were only slightly attenuated after adjustment for parental diagnoses and additional adjustment for level of education of the individual.

Our study adds to the body of existing knowledge by examining a broad spectrum of brain disorders and their associations with T2DM in a large population-based cohort. We also report novel findings on temporal ordering of co-occurrence, showing e.g. increased risk for anorexia nervosa and multiple sclerosis after T2DM, but not in the other direction. Replication of these findings is needed before any firm conclusions can be drawn. Future research on associations between T2DM and brain disorders should examine confounding and/or mediating effects of lifestyle factors and psychotropic medications. To elucidate the potential causal mechanisms, future epidemiological, genetic, and bioinformatic studies could assess to what extent brain disorders and T2DM are linked through shared familial risk.

## Supplementary Information

**Additional file 1: Supplementary Table 1.** Diagnostic classifications for definition of type 2 diabetes and selected brain disorders. **Supplementary Table 2.** Crude and adjusted estimates based on logistic regression analyses and temporally ordered Cox regression analyses. **Supplementary Table 3.** Temporally ordered analyses of sex-specific associations between diagnoses of brain disorders and type 2 diabetes. **Supplementary Table 4.** Adjusted estimates based on logistic regression analyses and temporally ordered Cox regression analyses without and with adjustment for highest attained level of education. **Supplementary Table 5.** Adjusted estimates based on logistic regression analysis applying the main and two alternative register-based T2DM definitions.

## Data Availability

The data that support the findings of this study are available from Statistics Denmark. The data access required the completion of a detailed application form from the Danish Data Protection Agency, the Danish National Board of Health and Statistics Denmark. For more information on accessing the data, see http://www.dst.dk.

## Abbreviations

ADHD: Attention-deficit/hyperactivity disorder
ASD: Autism spectrum disorder
ATC, OAD: Oral antidiabetic drug
OCD: Obsessive-compulsive disorder
T2DM: Type 2 diabetes mellitus
ALS: Amyothropic lateral sclerosis
ATC: Anatomical Therapeutic Chemical
CI: Confidence interval
HR: Hazard ratio
ICD: International Statistical Classification of Diseases
OAD: Oral antidiabetic drug
OR: Odds ratio
