# Supplementary material for: Temporally ordered associations between type 2 diabetes and brain disorders – a Danish register-based cohort study
Source: BMC Psychiatry. 2022 Aug 26;22:573. doi: 10.1186/s12888-022-04163-z (PMC9413891; doi:10.1186/s12888-022-04163-z)
Supplement: Supplementary file 1 — Additional file 1: Supplementary Table 1. Diagnostic classifications for definition of type 2 diabetes and selected brain disorders. Supplementary Table 2. Crude and adjusted estimates based on logistic regression analyses and temporally ordered Cox regression analyses. Supplementary Table 3. Temporally ordered analyses of sex-specific associations between diagnoses of brain disorders and type 2 diabetes. Supplementary Table 4. Adjusted estimates based on logistic regression analyses and temporally ordered Cox regression analyses without and with adjustment for highest attained level of education. Supplementary Table 5. Adjusted estimates based on logistic regression analysis applying the main and two alternative register-based T2DM definitions. [file 12888_2022_4163_MOESM1_ESM.docx]

## Supplementary material

Supplementary Table 1: Diagnostic classifications for definition of type 2 diabetes and selected brain disorders.

| **Disorder** | **ICD-10** | **ICD-8** | **Data source** |
| --- | --- | --- | --- |
| Diabetes mellitus type 2 | E11, O24.1 | 250 | DNPR |
| Diabetic complications |  |  |  |
| Diabetic cataract | H28.0 | 250.01 | DNPR |
| Diabetic retinopathy | H36.0 | 250.01 | DNPR |
| Diabetic traction detachment of retina | H33.4B |  | DNPR |
| Diabetic peripheral angiopathy | I79.2 | 250.04, 250.05 | DNPR |
| Diabetic mononeuropathy | G59.0 |  | DNPR |
| Diabetic polyneuropathy | G63.2 | 250.03 | DNPR |
| Autonomic neuropathy in endocrine and metabolic diseases | G99.0 |  | DNPR |
| Diabetic arthropathy | M14.2 |  | DNPR |
| Neuropathic arthropathy | M14.6 |  | DNPR |
| Glomerular disorders in diabetes mellitus | N08.3 | 250.02 | DNPR |
| Any psychiatric disorders (used for parental history) | F00-F99 | 295-315 | DPCRR |
| Psychiatric disorders |  |  |  |
| OCD | F42 | 300.39 | DPCRR, DNPR^a^ |
| ASD | F84 (excl. F84.2-4) | 299.00-299.03 | DPCRR, DNPR^a^ |
| Anorexia nervosa | F50.0, F50.1 | 306.50 | DPCRR, DNPR |
| Other eating disorder | F50.2, F50.3, F50.8, F50.9 | 306.58, 306.59 | DPCRR, DNPR |
| Substance use disorder | F10-F19, F10 (alcohol), F12 (cannabis), F11,F13-16,F18-19 (other illicit drugs) | 291.x9, 294.39, 303.x9, 303.20, 303.28, 303.90, 304.x9 | DPCRR, DNPR |
| Schizophrenia spectrum disorder | F20-F29 | 295.x9, 296.89, 297.x9, 298.29-298.99, 299.04, 299.05, 299.09, 301.83 | DPCRR |
| Major depressive disorder | F32-F33 | 296.09, 296.29, 298.09, 300.49 | DPCRR, DNPR |
| Bipolar disorder | F30-F31 | 296.19, 296.39, 298.19 | DPCRR |
| Anxiety disorder | F40.00-F41.19, F4200-F43.10, F93 | 300.09, 300.29, 300.39 | DPCRR |
| Personality disorder | F60 | 301.09, 301.29-301.99, 301.80, 301.81, 301.82, 301.84 | DPCRR |
| ADHD | F90, F98.8 | 308.1 | DPCRR, DNPR^a^ |
| Neurological disorders |  |  |  |
| Inflammatory brain diseases | G00-G09 | 320-324 | DPCRR, DNPR |
| Amyotrophic lateral sclerosis | G12.2 | 348.09 | DPCRR, DNPR |
| Epilepsy | G40-41 | 345, 309.49, 331.29 | DPCRR, DNPR |
| Multiple sclerosis | G35 | 340 | DPCRR, DNPR |
| Migraine | G43 | 346 | DPCRR, DNPR |

Abbreviations: ADHD: attention-deficit/hyperactivity disorder, ASD: Autism spectrum disorder, DNPR: The Danish National Patient Register, DPCRR: The Danish Psychiatric Central Research Register, OCD: Obsessive-compulsive disorder.

^a^ Diagnoses were obtained from DPCRR and from pediatric and neurology specialties in DNPR.

Supplementary table 2: Crude and adjusted estimates based on logistic regression analyses and temporally ordered Cox regression analyses.

|  | **Disorder and type 2 diabetes** | | | **Disorder and subsequent type 2 diabetes** | | | **Type 2 diabetes and subsequent disorder** | | |
| --- | --- | --- | --- | --- | --- | --- | --- | --- | --- |
| **Total N included = 1,883,198 ^a^** | **Cases** | **Crude OR** ^b^  **(95% CI)** | **Adjusted OR** ^c^  **(95% CI)** | **Cases** | **Crude HR** ^b^  **(95% CI)** | **Adjusted HR** ^c^  **(95% CI)** | **Cases** | **Crude HR** ^b^  **(95% CI)** | **Adjusted HR** ^c^  **(95% CI)** |
| **Psychiatric disorders** |  |  |  |  |  |  |  |  |  |
| OCD | 327 | 2.02 (1.81,2.26) | 1.98 (1.76,2.21) | 233 | 2.05 (1.80,2.33) | 2.00 (1.76,2.28) | 94 | 1.83 (1.49,2.25) | 1.79 (1.45,2.20) |
| ASD | 184 | 2.20 (1.89,2.55) | 2.17 (1.87,2.53) | 113 | 1.99 (1.66,2.40) | 1.97 (1.64,2.38) | 72 | 2.33 (1.83,3.05) | 2.38 (1.87,3.03) |
| Anorexia nervosa | 95 | 0.94 (0.77,1.15) | 0.94 (0.77,1.16) | 78 | 0.89 (0.71,1.11) | 0.89 (0.72,1.12) | 17 | 1.97 (1.21,3.19) | 1.97 (1.21,3.20) |
| Other eating disorder | 354 | 2.68 (2.40,2.99) | 2.64 (2.36,2.94) | 260 | 2.56 (2.26,2.89) | 2.50 (2.21,2.82) | 94 | 4.06 (3.29,5.00) | 3.92 (3.18,4.84) |
| Substance use disorder | 9,456 | 2.11 (2.06,2.16) | 2.05 (2.01,2.10) | 6,933 | 2.09 (2.04,2.14) | 2.03 (1.98,2.08) | 2,543 | 2.08 (1.99,2.16) | 2.02 (1.94,2.10) |
| Schizophrenia spectrum disorder | 3,082 | 2.82 (2.72,2.94) | 2.73 (2.63,2.84) | 2,740 | 2.91 (2.80,3.02) | 2.81 (2.70,2.92) | 351 | 2.02 (1.81,2.25) | 1.94 (1.74,2.16) |
| Major depressive disorder | 5,048 | 2.30 (2.23,2.37) | 2.25 (2.18,2.32) | 3,521 | 2.30 (2.23,2.38) | 2.25 (2.17,2.32) | 1,537 | 2.04 (1.94,2.15) | 1.99 (1.89,2.09) |
| Bipolar disorder | 936 | 2.20 (2.06,2.36) | 2.17 (2.02,2.32) | 658 | 2.30 (2.13,2.48) | 2.25 (2.08,2.43) | 284 | 1.85 (1.64,2.09) | 1.82 (1.61,2.06) |
| Anxiety disorder | 2,906 | 2.11 (2.03,2.19) | 2.04 (1.97,2.13) | 2,048 | 2.17 (2.07,2.26) | 2.10 (2.01,2.20) | 869 | 1.85 (1.72,1.98) | 1.79 (1.67,1.92) |
| Personality disorder | 4,075 | 2.51 (2.43,2.60) | 2.43 (2.35,2.52) | 3,597 | 2.51 (2.43,2.60) | 2.44 (2.36,2.52) | 482 | 2.08 (1.90,2.28) | 2.03 (1.85,2.22) |
| ADHD | 443 | 1.98 (1.80,2.19) | 1.88 (1.70,2.07) | 267 | 2.20 (1.95,2.47) | 2.07 (1.84,2.32) | 163 | 1.63 (1.39,1.91) | 1.55 (1.32,1.81) |
| **Neurological disorders** |  |  |  |  |  |  |  |  |  |
| Inflammatory brain diseases | 424 | 1.75 (1.58,1.93) | 1.73 (1.57,1.91) | 267 | 1.49 (1.32,1.68) | 1.48 (1.31,1.67) | 157 | 2.53 (2.15,2.98) | 2.51 (2.13,2.96) |
| Amyotrophic lateral sclerosis | 28 | 1.07 (0.73,1.57) | 1.07 (0.73,1.58) | 12 | 1.27 (0.72,2.23) | 1.25 (0.71,2.20) | 16 | 1.09 (0.66,1.81) | 1.09 (0.66,1.80) |
| Epilepsy | 2,238 | 1.71 (1.64,1.79) | 1.67 (1.60,1.75) | 1,653 | 1.60 (1.52,1.68) | 1.56 (1.49,1.64) | 589 | 2.30 (2.11,2.50) | 2.25 (2.06,2.44) |
| Multiple sclerosis | 375 | 1.02 (0.92,1.13) | 1.01 (0.91,1.12) | 236 | 0.90 (0.79,1.03) | 0.90 (0.79,1.02) | 139 | 1.36 (1.15,1.61) | 1.35 (1.14,1.60) |
| Migraine | 1,540 | 1.30 (1.23,1.37) | 1.29 (1.23,1.36) | 1,166 | 1.37 (1.29,1.45) | 1.37 (1.29,1.45) | 377 | - 1. 0.98,1.21) | 1.09 (0.98,1.20) |

**^a^** The two first analytic approaches includes the entire study population of 1,883,198 individuals. For the last approach (type 2 diabetes and subsequent brain disorder), only incident cases are included and hence the total number included in each analysis varies for each outcome, e.g. for OCD as the outcome (n = 1,880,480) and for ASD as the outcome (n=1,881,576).
^b^ Adjusted for sex and birth year.
^c^ Adjusted for sex, birth year, parental history of psychiatric disorders and type 2 diabetes. Estimates correspond to those reported and illustrated in Figures 1 and 2.
Abbreviations: ADHD: attention-deficit/hyperactivity disorder, ASD: Autism spectrum disorder, OCD: Obsessive-compulsive disorder.

Supplementary table 3: Temporally ordered analyses of sex-specific associations between diagnoses of brain disorders and type 2 diabetes

|  | **Disorder and subsequent type 2 diabetes** | | | | **Type 2 diabetes and subsequent disorder** | | | |
| --- | --- | --- | --- | --- | --- | --- | --- | --- |
|  | **Females**  (N=909,341) | | **Males**  (N=973,857) | | **Females**  (N=907,756 ^b^) | | **Males**  (N=973,138 ^b^) | |
|  | **Cases** | **Adjusted HR** ^a^  **(95% CI)** | **Cases** | **Adjusted HR** ^a^  **(95% CI)** | **Cases** | **Adjusted HR** ^a^  **(95% CI)** | **Cases** | **Adjusted HR** ^a^  **(95% CI)** |
| **Psychiatric disorders** |  |  |  |  |  |  |  |  |
| OCD | 136 | 2.14 (1.81,2.54) | 97 | 1.78 (1.46,2.17) | 51 | 1.78 (1.34,2.35) | 43 | 1.77 (1.30,2.41) |
| ASD | 32 | 2.05 (1.45,2.90) | 81 | 1.99 (1.60,2.47) | 21 | 2.29 (1.48,3.56) | 50 | 2.40 (1.80,3.21) |
| Anorexia nervosa^c^ | NA | NA | NA | NA | NA | NA | NA | NA |
| Other eating disorder | 238 | 2.26 (1.99,2.57) | 22 | 3.49 (2.30,5.30) | 79 | 3.66 (2.91,4.60) | 15 | 6.06 (3.48,10.55) |
| Substance use disorder | 2,079 | 2.18 (2.08,2.28) | 3,893 | 1.97 (1.91,2.03) | 917 | 2.14 (2.00,2.28) | 1,606 | 1.97 (1.87,2.07) |
| Schizophrenia spectrum disorder | 1,205 | 3.42 (3.22,3.62) | 1,535 | 2.48 (2.36,2.61) | 153 | 2.28 (1.94,2.68) | 189 | 1.77 (1.53,2.05) |
| Major depressive disorder | 2,014 | 2.45 (2.34,2.57) | 1,504 | 2.01 (1.91,2.12) | 734 | 1.94 (1.80,2.09) | 793 | 2.00 (1.86,2.15) |
| Bipolar disorder | 365 | 2.55 (2.30,2.83) | 291 | 1.97 (1.75,2.21) | 132 | 1.75 (1.47,2.09) | 146 | 1.87 (1.58,2.21) |
| Anxiety disorder | 1,140 | 2.17 (2.04,2.30) | 906 | 2.01 (1.88,2.15) | 440 | 1.86 (1.69,2.04) | 418 | 1.71 (1.55,1.89) |
| Personality disorder | 2,056 | 2.83 (2.70,2.96) | 1,535 | 2.05 (1.95,2.16) | 258 | 2.07 (1.83,2.34) | 220 | 1.98 (1.73,2.27) |
| ADHD | 105 | 2.39 (1.98,2.90) | 177 | 1.95 (1.68,2.26) | 72 | 1.76 (1.39,2.23) | 89 | 1.40 (1.14,1.74) |
| **Neurological disorder** |  |  |  |  |  |  |  |  |
| Inflammatory brain diseases | 115 | 1.68 (1.40,2.01) | 152 | 1.36 (1.16,1.60) | 50 | 2.09 (1.57,2.78) | 107 | 2.79 (2.28,3.41) |
| Amyotrophic lateral sclerosis^c^ | NA | NA | NA | NA | NA | NA | NA | NA |
| Epilepsy | 711 | 1.68 (1.56,1.81) | 938 | 1.48 (1.39,1.58) | 227 | 2.57 (2.24,2.94) | 358 | 2.05 (1.84,2.29) |
| Multiple sclerosis | 148 | 0.94 (0.80,1.11) | 88 | 0.84 (0.68,1.03) | 75 | 1.21 (0.97,1.53) | 64 | 1.54 (1.20,1.98) |
| Migraine | 784 | 1.42 (1.32,1.52) | 382 | 1.28 (1.16,1.41) | 281 | 1.18 (1.05,1.33) | 93 | 0.87 (0.71,1.07) |

^a^ Adjusted for birth year, parental history of type 2 diabetes and psychiatric disorders.
^b^ As only incident cases are included in this analysis, the study population varies for each outcome.
c Cases in males or females were too few to be reported, according to regulations at Statistics Denmark and CIRRAU.
Abbreviations: ADHD: attention-deficit/hyperactivity disorder, ASD: Autism spectrum disorder, CIRRAU: Centre for Integrated Register-based Research at Aarhus University, OCD: Obsessive-compulsive disorder.

Supplementary Table 4: Adjusted estimates based on logistic regression analyses and temporally ordered Cox regression analyses without and with adjustment for highest attained level of education.

|  | **Disorder and type 2 diabetes** | | | **Disorder and subsequent type 2 diabetes** | | | **Type 2 diabetes and subsequent disorder** | | |
| --- | --- | --- | --- | --- | --- | --- | --- | --- | --- |
| **Total N included = 1,883,198 ^a^** | **Cases** | **Adjusted OR** ^b^  **(95% CI)** | **Adjusted OR** ^c^  **(95% CI)** | **Cases** | **Adjusted HR** ^b^  **(95% CI)** | **Adjusted HR** ^c^  **(95% CI)** | **Cases** | **Adjusted HR** ^b^  **(95% CI)** | **Adjusted HR** ^c^  **(95% CI)** |
| **Psychiatric disorders** |  |  |  |  |  |  |  |  |  |
| OCD | 327 | 1.98 (1.76,2.21) | 1.74 (1.55,1.95) | 233 | 2.00 (1.76,2.28) | 1.81 (1.59,2.06) | 94 | 1.79 (1.45,2.20) | 1.57 (1.27,1.93) |
| ASD | 184 | 2.17 (1.87,2.53) | 1.54 (1.32,1.79) | 113 | 1.97 (1.64,2.38) | 1.39 (1.15,1.67) | 72 | 2.38 (1.87,3.03) | 1.80 (1.42,2.30) |
| Anorexia nervosa | 95 | 0.94 (0.77,1.16) | 0.87 (0.71,1.07) | 78 | 0.89 (0.72,1.12) | 0.87 (0.69,1.08) | 17 | 1.97 (1.21,3.20) | 1.79 (1.10,2.90) |
| Other eating disorder | 354 | 2.64 (2.36,2.94) | 2.44 (2.19,2.73) | 260 | 2.50 (2.21,2.82) | 2.38 (2.11,2.69) | 94 | 3.92 (3.18,4.84) | 3.54 (2.86,4.37) |
| Substance use disorder | 9,456 | 2.05 (2.01,2.10) | 1.70 (1.66,1.74) | 6,933 | 2.03 (1.98,2.08) | 1.68 (1.64,1.72) | 2,543 | 2.02 (1.94,2.10) | 1.69 (1.62,1.76) |
| Schizophrenia spectrum disorder | 3,082 | 2.73 (2.63,2.84) | 2.21 (2.13,2.30) | 2,740 | 2.81 (2.70,2.92) | 2.34 (2.25,2.43) | 351 | 1.94 (1.74,2.16) | 1.61 (1.44,1.80) |
| Major depressive disorder | 5,048 | 2.25 (2.18,2.32) | 1.98 (1.92,2.04) | 3,521 | 2.25 (2.17,2.32) | 2.02 (1.95,2.09) | 1,537 | 1.99 (1.89,2.09) | 1.77 (1.68,1.86) |
| Bipolar disorder | 936 | 2.17 (2.02,2.32) | 1.95 (1.82,2.09) | 658 | 2.25 (2.08,2.43) | 2.08 (1.92,2.24) | 284 | 1.82 (1.61,2.06) | 1.66 (1.47,1.87) |
| Anxiety disorder | 2,906 | 2.04 (1.97,2.13) | 1.74 (1.68,1.81) | 2,048 | 2.10 (2.01,2.20) | 1.83 (1.75,1.92) | 869 | 1.79 (1.67,1.92) | 1.54 (1.44,1.65) |
| Personality disorder | 4,075 | 2.43 (2.35,2.52) | 1.98 (1.91,2.04) | 3,597 | 2.44 (2.36,2.52) | 2.03 (1.96,2.10) | 482 | 2.03 (1.85,2.22) | 1.69 (1.55,1.86) |
| ADHD | 443 | 1.88 (1.70,2.07) | 1.37 (1.24,1.51) | 267 | 2.07 (1.84,2.32) | 1.52 (1.35,1.71) | 163 | 1.55 (1.32,1.81) | 1.17 (1.00,1.37) |
| **Neurological disorder** |  |  |  |  |  |  |  |  |  |
| Inflammatory brain diseases | 424 | 1.73 (1.57,1.91) | 1.62 (1.46,1.79) | 267 | 1.48 (1.31,1.67) | 1.41 (1.25,1.59) | 157 | 2.51 (2.13,2.96) | 2.39 (2.03,2.82) |
| Amyotrophic lateral sclerosis | 28 | 1.07 (0.73,1.58) | 1.06 (0.72,1.56) | 12 | 1.25 (0.71,2.20) | 1.26 (0.71,2.22) | 16 | 1.09 (0.66,1.80) | 1.09 (0.66,1.80) |
| Epilepsy | 2,238 | 1.67 (1.60,1.75) | 1.41 (1.35,1.48) | 1,653 | 1.56 (1.49,1.64) | 1.33 (1.26,1.39) | 589 | 2.25 (2.06,2.44) | 1.97 (1.81,2.14) |
| Multiple sclerosis | 375 | 1.01 (0.91,1.12) | 0.99 (0.89,1.10) | 236 | 0.90 (0.79,1.02) | 0.88 (0.77,1.00) | 139 | 1.35 (1.14,1.60) | 1.33 (1.12,1.58) |
| Migraine | 1,540 | 1.29 (1.23,1.36) | 1.24 (1.18,1.31) | 1,166 | 1.37 (1.29,1.45) | 1.32 (1.24,1.40) | 377 | 1.09 (0.98,1.20) | 1.06 (0.95,1.17) |

^a^ The two first analytic approaches includes the entire study population of 1,883,198 individuals. For the last approach (type 2 diabetes and subsequent brain disorder), only incident cases are included and hence the total number included in each analysis varies for each outcome, e.g. for OCD as the outcome (n = 1,880,480) and for ASD as the outcome (n=1,881,576).
^b^ Adjusted for sex, birth year, parental history of type 2 diabetes and psychiatric disorders.
^c^ Additionally adjusted for highest obtained education level by age 30 years (not completed, low, intermediate, high).
Abbreviations: ADHD: attention-deficit/hyperactivity disorder, ASD: Autism spectrum disorder, OCD: Obsessive-compulsive disorder.

Supplementary table 5: Adjusted estimates based on logistic regression analysis applying the main and two alternative register-based T2DM definitions

|  | **Main and alternative register-based T2DM definitions** | | | | | |
| --- | --- | --- | --- | --- | --- | --- |
|  | **Main (at least one OAD prescription or diagnosis)**  (n = 67,660 ) | | **Requiring at least two OAD prescriptions**  (n = 64,701) | | **Clinical diagnosis ^c^ only**  (n = 41,498) | |
| **Total N included = 1,883,198** | Cases | Adjusted OR ^a^ (95% CI) | Cases | Adjusted OR ^a^ (95% CI) | Cases | Adjusted OR ^a^ (95% CI) |
| **Psychiatric disorders** |  |  |  |  |  |  |
| OCD | 327 | 1.98 (1.76,2.21) | 314 | 2.00 (1.78,2.24) | 219 | 2.12 (1.85,2.43) |
| ASD | 184 | 2.17 (1.87,2.53) | 176 | 2.18 (1.87,2.55) | 120 | 2.26 (1.88,2.72) |
| Anorexia nervosa | 95 | 0.94 (0.77,1.16) | NA ^b^ | NA ^b^ | 77 | 1.23 (0.98,1.55) |
| Other eating disorder | 354 | 2.64 (2.36,2.94) | 334 | 2.64 (2.36,2.95) | 237 | 2.73 (2.39,3.12) |
| Substance use disorder | 9,456 | 2.05 (2.01,2.10) | 9,107 | 2.06 (2.01,2.11) | 6,820 | 2.44 (2.38,2.51) |
| Schizophrenia spectrum disorder | 3,082 | 2.73 (2.63,2.84) | 2,950 | 2.72 (2.62,2.83) | 1,951 | 2.70 (2.57,2.83) |
| Major depressive disorder | 5,048 | 2.25 (2.18,2.32) | 4,828 | 2.25 (2.18,2.32) | 3,377 | 2.43 (2.34,2.52) |
| Bipolar disorder | 936 | 2.17 (2.02,2.32) | 897 | 2.17 (2.02,2.33) | 598 | 2.21 (2.03,2.40) |
| Anxiety disorder | 2,906 | 2.04 (1.97,2.13) | 2,773 | 2.04 (1.96,2.13) | 1,903 | 2.15 (2.05,2.26) |
| Personality disorder | 4,075 | 2.43 (2.35,2.52) | 3,907 | 2.44 (2.36,2.53) | 2,735 | 2.60 (2.50,2.71) |
| ADHD | 443 | 1.88 (1.70,2.07) | 411 | 1.83 (1.65,2.02) | 275 | 1.85 (1.64,2.09) |
| **Neurological disorders** |  |  |  |  |  |  |
| Inflammatory brain diseases | 424 | 1.73 (1.57,1.91) | 408 | 1.74 (1.57,1.93) | 334 | 2.22 (1.98,2.48) |
| Amyothrophic lateral sclerosis | 28 | 1.07 (0.73,1.58) | NA ^b^ | NA ^b^ | 20 | 1.25 (0.80,1.96) |
| Epilepsy | 2,238 | 1.67 (1.60,1.75) | 2,151 | 1.68 (1.60,1.75) | 1,609 | 1.94 (1.85,2.05) |
| Multiple Sclerosis | 375 | 1.01 (0.91,1.12) | 361 | 1.02 (0.92,1.13) | 254 | 1.13 (0.99,1.28) |
| Migraine | 1,540 | 1.29 (1.23,1.36) | 1,450 | 1.28 (1.21,1.35) | 1,007 | 1.39 (1.30,1.48) |

^a^ Adjusted for sex, birth year, parental history of type 2 diabetes and psychiatric disorders.
^b^ Differences in number of exposed cases between our main and second definition were too few for results to be reported, according to regulations at Statistics Denmark and CIRRAU.
^c^ Includes hospital diagnoses of T2DM (approximately 90% of cases) and diabetes-related complications as listed in supplementary table 1.
Abbreviations: ADHD: attention-deficit/hyperactivity disorder, ASD: Autism spectrum disorder, CIRRAU: Centre for Integrated Register-based Research at Aarhus University, OAD: Oral anti-diabetics, OCD: Obsessive-compulsive disorder.
